# Radio-Frequency Conductivity Characteristics and Corresponding Mechanism of Graphene/Copper Multilayer Structures

**DOI:** 10.3390/ma17122999

**Published:** 2024-06-19

**Authors:** Chongxiao Guo, Jian Song, Jiamiao Ni, Yue Liu, Tongxiang Fan

**Affiliations:** State Key Lab of Metal Matrix Composites, School of Materials Science and Engineering, Shanghai Jiao Tong University, Shanghai 200240, China; guo083013@sjtu.edu.cn (C.G.); jsong18@sjtu.edu.cn (J.S.); nijiamiao@sjtu.edu.cn (J.N.)

**Keywords:** graphene/copper multilayer, radio-frequency conductivity, scanning microwave impedance microscopy, scattering mechanism

## Abstract

High-radio-frequency (RF) conductivity is required in advanced electronic materials to reduce the electromagnetic loss and power dissipation of electronic devices. Graphene/copper (Gr/Cu) multilayers possess higher conductivity than silver under direct current conditions. However, their RF conductivity and detailed mechanisms have rarely been evaluated at the micro scale. In this work, the RF conductivity of copper–copper (P-Cu), monolayer-graphene/copper (S-Gr/Cu), and multilayer-graphene/copper (M-Gr/Cu) multilayer structures were evaluated using scanning microwave impedance microscopy (SMIM) and dielectric resonator technique. The results indicated that the order of RF conductivity was M-Gr/Cu < P-Cu < S-Gr/Cu at 3 GHz, contrasting with P-Cu < M-Gr/Cu < S-Gr/Cu at DC condition. Meanwhile, the same trend of M-Gr/Cu < P-Cu < S-Gr/Cu was also observed using the dielectric resonator technique. Based on the conductivity-related Drude model and scattering theory, we believe that the microwave radiation can induce a thermal effect at S-Gr/Cu interfaces, leading to an increasing carrier concentration in S-Gr. In contrast, the intrinsic defects in M-Gr introduce additional carrier scattering, thereby reducing the RF conductivity in M-Gr/Cu. Our research offers a practical foundation for investigating conductive materials under RF conditions.

## 1. Introduction

Metallic materials, particularly copper (Cu), have attracted significant attention for mitigating electromagnetic loss and power dissipation under high-radio-frequency (RF) conditions owing to their high-conductivity properties [1,2,3]. Enhancing the electrical conductivity of Cu has become a long-standing topic in meeting the requirements of high-throughput information processing that is driven by the development of the modern electronics industry [4]. Aiming at enhancing the conductivity of Cu materials, much research has focused on processing optimizations and microstructure modulations. Lu et al. creatively synthesized pure copper samples with a high density of nanoscale growth twins. They showed a tensile strength over 1000 MPa, while retaining an electrical conductivity comparable to that of pure copper (96.9% of International Annealed Cu Standard (IACS)) [5]. Gurminder Singh et al. achieved rapid preparation of copper conductive specimens by technological hybridization of three-dimensional printing technology and ultrasonic-assisted pressure-less sintering, with a conductivity of 84.59% IACS. It was found that the sintering temperature and ultrasonic power percentage intensity are the most important parameters affecting the conductivity of sintered samples [6]. Sun et al. developed annealed oxygen-free copper and discovered that the increase in electrical conductivity (~100% IACS) is mainly due to the decrease in dislocation density and the transformation of grain boundaries from a nonequilibrium state to an equilibrium state [7]. The above studies have achieved encouraging results in exploring copper conductivity; however, achieving conductivity surpassing that of single-crystal Cu remains elusive (~101.5% IACS) [8].

The advent of graphene (Gr) provides a dependable strategy to enhance the conductivity of metallic materials [9,10]. Unlike metallic materials with high carrier concentration and low carrier mobility characteristics, Gr, with its high carrier mobility, is anticipated to compensate for the deficiencies of metallic materials in electrical conductivity, thereby yielding materials with superior conductivity compared to single-crystal Cu [11,12]. Specifically, Xiong et al. fabricated Gr on the surface of Cu films, where the electronic interactions between Gr and Cu led to the amalgamation of ultra-high carrier mobility and high carrier concentration features of metallic Cu, culminating in a direct current (DC) conductivity surpassing that of single-crystal Cu and silver (Ag), reaching 117% IACS [13]. Considering the presence of the skin effect at high-radio-frequency (RF) fields, it can be expected that the electrical properties of Gr and the electronic interaction at the Gr/Cu interface will be amplified and thus may dominate the RF conductivity of Gr/Cu [14]. For instance, Wu et al. tested the conductivity of monolayer Gr at 40 GHz through the construction of a coplanar waveguide transmission line structure. The results revealed a distinct frequency dependency of Gr’s conductivity and sheet resistance, with conductivity decreasing as frequency increased [15]. Jerzy Krupka et al. studied epitaxial Gr of various thicknesses deposited on semi-insulating silicon carbide at microwave frequencies (10–13.22 GHz) using resonators. The investigation revealed that the conductivity of Gr exhibited a trend of initially decreasing and then increasing with increasing thickness [16]. Despite efforts to study the conductivity of pristine Gr under RF conditions, characterizing the electronic interactions at the Gr/Cu interface is still needed to provide insight into the design and fabrication of Gr/Cu materials with desired RF conductivity. However, current RF conductivity assessments primarily employ conventional methods, such as various resonators and transmission lines, which fail to capture the impact of electronic interactions at the Gr/Cu interface on RF conductivity. This is primarily attributed to the electronic interactions between Gr and Cu occurring at sub-micro or even nanometer scales, where conventional testing methods such as resonant cavities and SubMiniature version A (SMA) connections are unable to achieve the required resolution for RF conductivity at the micrometer scale [17,18].

Scanning microwave impedance microscopy (SMIM) has garnered widespread popularity due to its capability to assess RF field conductivity with ultra-high resolution. Liu employed scanning microwave near-field microscopy to identify defects or microstructures resulting from the growth and processing of Ag/Cr multilayer film microwave devices, achieving a spatial resolution of less than 5 μm [19]. Tselev et al. determined the surface resistance of Gr films deposited on various substrates using scanning microwave near-field microscopy and numerical simulation. The investigation revealed that the impact of surface morphology on the surface resistance of Gr films could be resolved to 50 nm [20]. Pushkarev et al. examined the photoconductivity of GaAs nanorod arrays, each with a width of 450 nm, using SMIM. The study unveiled that energy band bending near the nanorods’ surface resulted in prolonged electron localization [21]. These studies demonstrate the feasibility of assessing the local RF conductivity of Gr/Cu multilayer structures using SMIM and analyzing the factors influencing RF conductivity.

Nevertheless, it should be noted that the influence of microstructure characteristics, such as the number of layers and microscopic defects of Gr, cannot be neglected when testing RF conductivity at the Gr/Cu interface using SMIM. Lin found that AA-stacked multilayer Gr may have higher mobility than monolayer Gr if the Gr has a large impurity density through terahertz optical modeling. Additionally, Lin et al. determined that the effective scattering rate of Gr varies with frequency and cannot be fully captured by a simple Drude model [22]. Fonseca et al. examined the scattering of Gr by topological defects, including holes, pentagons, and heptagons. The findings indicated that the impact of a low concentration of holes on resistivity was negligible. Moreover, the introduction of topological defects in the form of pentagons or heptagons leads to defect scattering of fermionic currents [23]. Talanov et al. examined the surface resistance of Gr with various thicknesses using SMIM. The study revealed that the energy band spectrum near the charge neutral point undergoes significant alterations due to the varied stacking of Gr with differing layers, resulting in increased scattering time with increasing thicknessand thickness-dependent resistance of Gr flakes [24].

According to the above research, it can be found that SMIM has a precise resolution in determining the RF conductivity at micrometer/nanoscale range. In addition, existing research has also suggested that microstructure characteristics (such as the number of layers and micro defects of Gr) have an undeniable impact on the RF conductivity of Gr. However, there are still some issues that need to be further discussed: (a) the differences in the conductivity of Gr/Cu multilayer structures under DC and RF conditions; (b) the mechanism causing the differences of conductivity in RF and DC conditions; and (c) the factors by which Gr characteristics affect the RF conductivity of Gr/Cu multilayer structures.

In this study, Gr/Cu multilayer structures were examined at RF conditions using SMIM to assess the impact of electronic interactions among monolayer Gr, multilayer Gr, and Cu on their RF conductivity. The findings reveal that the RF conductivity of monolayer Gr/Cu (S-Gr/Cu) structures surpasses that of pure Cu (P-Cu) under RF conditions, whereas that of multilayer Gr/Cu (M-Gr/Cu) is inferior to pure Cu, contrary to the results observed under DC conditions. Examination of carrier concentration, mobility, and structural features of multilayer Gr revealed that the intrinsic defects in multilayer Gr can introduce an additional pinning effect on the Fermi energy level of Gr and degrade the electronic interaction of M-Gr/Cu interface, thereby resulting in lower RF conductivity of M-Gr/Cu compared to pure Cu counterpart. Our research can provide reference for analyzing the RF conductivity of reinforced metal multilayer structures with Gr.

## 2. Experimental Methods

### 2.1. Fabrication of Gr/Cu Multilayer Structures

**Figure 1:**
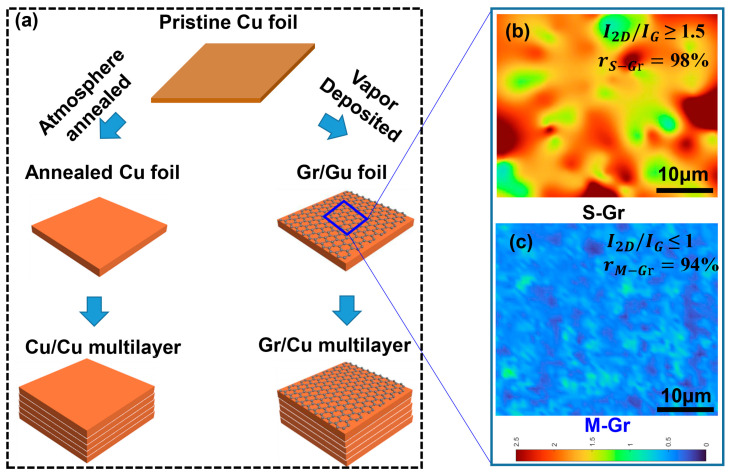
Cu/Cu and Gr/Cu multilayer structure preparation process and Gr Raman mapping result: (**a**) Cu/Cu and Gr/Cu multilayer structure preparation process, (**b**) S-Gr Raman mapping result, (**c**) M-Gr Raman mapping result.

Figure 1a illustrates the process for preparing Cu/Cu and Gr/Cu multilayer structures. Initially, monolayer Cu films were annealed under a hydrogen atmosphere for 30 min. Next, separate processes of hydrogen annealing with 30 min and methane-assisted chemical vapor deposition (CVD) with 30 min were performed to obtain annealed Cu film and Gr/Cu film, respectively. By fixing the gas flux of hydrogen (200 sccm) and methane (6 sccm) by adjusting the pressure of the tube furnace to 1 Torr and 250 Torr, respectively, different layers of graphene can be deposited on the copper surface. Finally, the annealed Cu and Gr/Cu films were rolled into Cu/Cu and Gr/Cu multilayers using self-developed roll-to-roll and hot isostatic pressing techniques. The details of the preparation process and samples can be found in reference [25].

### 2.2. Characterizations of the Gr/Cu Multilayer Structures

Raman spectroscopy, utilizing a 532 nm Ar^+^ laser and confocal microscopic setup (IinVia Reflex, Renishaw, London, UK), was employed to analyze the number of layers and coverage of Gr deposited on the surface of Cu films at room temperature. The laser spot size was approximately 1 μm. By scanning a specific area using Raman spectroscopy and analyzing the characteristic peak position and intensity, the number and coverage of graphene layers as well as the presence of defects can be determined.

The resolution of the Raman spectra was approximately 1 μm, matching that of the laser spot size. Subsequently, a scanning electron microscope (SEM; RISE-Magna, TESCAN, Brno, Czech) was employed to examine the microscopic features of the Gr/Cu interface to mitigate the potential damage caused by hot isostatic pressing.

### 2.3. RF Conductivity Measurements of the Gr/Cu Multilayer Structures

The RF conductivity of the Cu/Gr multilayer structure was determined using the SMIM test system (PrimeNano Company, Santa Clara, CA, USA). This system consists of a vector network analyzer (VNA), a coaxial cavity with sharp probes, an x–y two-dimensional motor stage, and a computer. The x–y two-dimensional motor stage holds the sample, and the scanning speed is controlled by the computer with a precision of 1 μm. The movement of the probe along the z-axis can be controlled in 20 μm increments [26]. An in-depth explanation of the derivation of the RF conductivity of the material as determined by the SMIM system is provided in the subsequent section.

To address potential issues with SMIM test results, we conducted measurements of the RF conductivity of P-Cu, S-Gr/Cu, and M-Gr/Cu thin films at 3.1 GHz and 8.2 GHz using a dielectric resonant cavity test system. The system comprises an aluminum alloy resonant cavity, a vector network analyzer, and a resonant cavity coupling device. Two microwave coupling lines are linked to the electromagnetic field within the cavity in a loop coupling manner, which is connected to the network analyzer as the input and output for the microwave signals, respectively [27]. The subsequent section will elaborate on the methodology used to derive the RF conductivity of the material employing the dielectric resonant cavity system.

## 3. Results and Discussion

### 3.1. Raman Spectra of Deposited Gr

Previous studies have shown that the number of layers and the coverage of Gr significantly affect the conductivity of Gr/Cu multilayer structures. To verify the coverage and number of layers of Gr, random positions (as depicted by the blue box in Figure 1a) were selected for Raman mapping, with the results are displayed in Figure 1b, c. The ratio of the 2D peak (I_2D_) to the G peak (I_G_) in the Raman mapping results was utilized to identify the number of Gr layers within an approximately 40 μm × 40 μm area. By calculating the ratio of I_2D_/I_G_ within specific intervals as a proportion of the total number of tested regions, the coverage of the corresponding layers was determined [28]. Table 1 presents the distribution of I_2D_/I_G_ ratios calculated from the Raman mapping results. The proportions of I_2D_/I_G_ ratios within the ranges 1.5–2.5, 1–1.5, and 0–1 for Gr deposited at 1 Torr are 98.1%, 1.3%, and 0.2%, respectively. The proportions of I_2D_/I_G_ ratios within the ranges 1.5–2.5, 1–1.5, and 0–1 for Gr deposited at 250 Torr are 2.1%, 3.7%, and 94.2%, respectively. The different colors in Figure 1b, c represent the corresponding I_2D_/I_G_ ratios, which fluctuate between 0 and 2.5. As shown in Figure 1b, the ratio of I_2D_/I_G_ exceeds 1.5, indicating the presence of monolayer Gr (S-Gr). In addition, the colored mapping shows that the coverage of S-Gr is approximately 98%. Similarly, the coverage of multilayer graphene (M-Gr) in Figure 1c is approximately 94%. The presence of M-Gr can be attributed to the gradual increased growth pressure, resulting in the diffusion of carbon atoms beneath the Gr layer, followed by nucleation and growth [29]. These results confirm the successful synthesis of S-Gr and M-Gr with comparable coverage on Cu.

### 3.2. RF Conductivity of Gr/Cu

**Figure 2:**
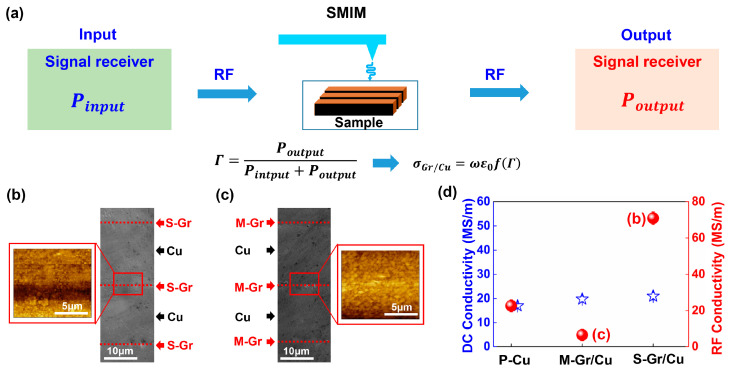
Principle of SMIM test and conductivity test results as well as microscopic morphology of the test area: (**a**) Principle of SMIM test process and conductivity derivation, (**b**) Microscopic morphology and voltage distribution of the RF conductivity test area of S-Gr/Cu (**c**) Microscopic morphology and voltage distribution of the RF conductivity test area of M-Gr/Cu morphology and voltage distribution in the M-Gr/Cu RF conductivity test area, (**d**) DC and RF conductivities of P-Cu, M-Gr/Cu, and S-Gr/Cu.

After determining the number of layers and coverage of deposited Gr, SMIM was utilized to assess the RF conductivity at the Gr/Cu interface. The testing principle is shown in Figure 2a. Microwave signals are transmitted through the vector network analyzer, traversed by an isolator and circulator delineated within the light green box on the left side of Figure 2a. Subsequently, these signals are conveyed to the SMIM probe via a signal cable with a specific impedance. The probe emitted microwave signals towards the sample, inducing emission, while the detector linked to the circulator measured the reflected microwave signal captured at the probe’s tip, as depicted at the top of Figure 2a. Finally, the voltage data are determined after converting the reflected microwave signal. The voltage data acquired from the SMIM test represent raw data converted from the signal reflected and received by the detector. To deduce the conductivity data from the voltage data, the subsequent procedure is employed [30,31,32]: The reflected voltage V output from the SMIM is related to the absolute value of the square of the reflection coefficient $\left|\Gamma_{s}\right|$, as follows:

(1) $$V=k_{0}{\left|\Gamma_{s}\right|}^{2}+b_{0}$$

Two conductivity samples with known values can be utilized for calibration to determine the values of the constants $k_{0}$ and $b_{0}$. For the good conductors prepared in this experiment, the surface reflection coefficient is correlated with the impedance of the testing system. This relationship can be expressed as follows:

(2) $$\Gamma_{s}=\frac{Z_{s}-Z_{0}}{Z_{s}+Z_{0}}$$

where $Z_{0}$ is the initial characteristic impedance of the system. And $Z_{s}$ is the surface impedance directly related to the conductivity of the system, which can be obtained by Equation (3):

(3) $$Z_{s}=\sqrt{\frac{i\omega \mu_{0}}{\sigma +i\omega \varepsilon }}$$

where ω is the testing frequency, $\mu_{0}$ is the permeability of free space, and ε is the dielectric constant. By solving Equations (2) and (3) simultaneously, the $\Gamma_{s}$ can be expressed as follows:

(4) $$\left|\Gamma_{s}\right|=\left|\frac{1-\sqrt{\frac{\sigma }{j\omega \varepsilon_{0}}}}{1+\sqrt{\frac{\sigma }{j\omega \varepsilon_{0}}}}\right|$$

where $\varepsilon_{0}$ and σ represent the dielectric constant of free space and the conductivity of the tested material, respectively, and ω is the angular frequency of the microwave. Similar equations can be established for semiconductors or insulating materials. The conductivity can then be determined from Equation (4), as follows:

(5) $$\sigma =\omega \varepsilon_{0}\frac{4{\left|\Gamma_{s}\right|}^{2}-{\left({\left|\Gamma_{s}\right|}^{2}+1\right)}^{2}}{\left({\left|\Gamma_{s}\right|}^{2}+1\right)\sqrt{4{\left|\Gamma_{s}\right|}^{2}-{\left({\left|\Gamma_{s}\right|}^{2}-1\right)}^{2}}-4{\left|\Gamma_{s}\right|}^{2}}$$

Based on the constants $k_{0}$ and $b_{0}$ acquired from Equation (1), the conductivity of the sample under measurement can be calculated from the measured voltage data using Equation (5).

The results of SMIM testing for RF conductivity at the interfaces of S-Gr/Cu and M-Gr/Cu are depicted in Figure 2b,c. To mitigate the influence of interfacial conditions on the RF conductivity of the materials, the interfacial morphologies of S-Gr/Cu and M-Gr/Cu were individually examined, as depicted in the SEM images in Figure 2b,c. Each SEM morphology included three Gr/Cu interfaces, and the Cu foils had a thickness of approximately 20 μm after thermal isostatic pressing, with no discernible holes or cracks at the interfaces. The red box in the SEM image in Figure 2b,c delineates the scanning area of the SMIM, with the corresponding voltage distribution map obtained post-SMIM testing displayed on the left. In the case of S-Gr/Cu, the voltage values within 2 μm of the Gr/Cu interface are lower than those in the Cu matrix. Conversely, at the M-Gr/Cu interface, the voltage values are higher than those in the surrounding Cu matrix, as depicted in Figure 2c.

By combining the voltage values measured by SMIM with the calculations of Equations (1)–(5), the RF conductivity of P-Cu, S-Gr/Cu, and M-Gr/Cu can be obtained, represented by the red spheres shown in Figure 2d. The RF conductivity order of the three materials is M-Gr/Cu < P-Cu < S-Gr/Cu. Meanwhile, the DC conductivity of P-Cu, S-Gr/Cu, and M-Gr/Cu multilayer structures is represented by blue pentagrams in Figure 2d, compared to the current study. Table 2 presents the specific values of the electrical conductivities of P-Cu, M-Gr/Cu, and S-Gr/Cu under DC and SMIM conditions. The findings consistently indicate that S-Gr/Cu shows the highest conductivity, reaching 21 MS/m under DC conditions and 71.1 MS/m under RF conditions. It is noteworthy that the relative conductivity between M-Gr/Cu and P-Cu exhibits an inverse pattern under DC and RF conditions. Under DC conditions, M-Gr/Cu exhibits higher conductivity than P-Cu, whereas the RF conductivity of M-Gr/Cu is lower than that of the P-Cu counterpart.

**Figure 3:**
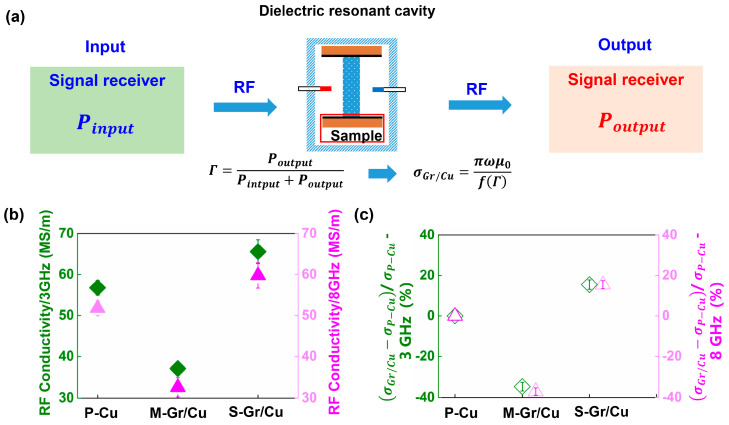
Comparison of conductivity at different frequencies for P-Cu, S-Gr/Cu, and M-Gr/Cu, and the difference in RF conductivity between Gr/Cu and P-Cu using dielectric resonance cavity: (**a**) Principle of dielectric resonance cavity, (**b**) RF conductivity at different frequencies for P-Cu, S-Gr/Cu, and M-Gr/Cu, (**c**) Difference in RF conductivity between S-Gr/Cu and M-Gr/Cu and P-Cu at different frequencies.

To mitigate any potential uncertainties in the SMIM results, Figure 3 illustrates the measured RF conductivities of these films at various frequencies (ω = 3.1, 8.2 GHz) using the dielectric resonance cavity test technique. The principle of this testing technique is depicted in Figure 3a. The process entails inputting a high-frequency signal from a source and outputting it through the receiver post-passage through the Gr/Cu interface (indicated by the red box in Figure 3a). The derivation of the RF conductivity of the test sample is also entirely dependent on the ratio of the input power to the total power (reflection coefficient $\left|\Gamma_{s}\right|$). The $\left|\Gamma_{s}\right|$ of the dielectric resonance cavity is linked to the disparity between the quality factor of the cavity with and without loading, which arises from the surface resistance of the sample. Finally, the RF conductivity of the sample can be inferred directly from its surface resistance.

Figure 3b illustrates the variation in RF conductivity with various frequencies for P-Cu, S-Gr/Cu, and M-Gr/Cu under conditions of 3.1 GHz and 8.2 GHz using dielectric resonator cavity testing. The RF conductivity of the three materials slightly decreases with increasing test frequency. Table 3 displays the specific values of RF conductivity of P-Cu, M-Gr/Cu, and S-Gr/Cu. At 3.1 GHz, the RF conductivities for P-Cu, S-Gr/Cu, and M-Gr/Cu are (56.8 ± 1.8) MS/m, (37.1 ± 1.4) MS/m, and (65.6 ± 2.9) MS/m, respectively. At 8.2 GHz, the RF conductivities for P-Cu, S-Gr/Cu, and M-Gr/Cu are (51.8 ± 2.5) MS/m, (31.6 ± 2.3) MS/m, and (59.8 ± 3.1) MS/m, respectively. The decrease in RF conductivity with increasing frequency is approximately 10%, thereby ruling out the interference of testing errors (below 5%). Importantly, the sequential order of RF conductivity among M-Gr/Cu, P-Cu, and S-Gr/Cu remains consistent at different frequencies (ω = 3.1, 8.2 GHz). Furthermore, taking the RF conductivity of P-Cu at different frequencies as the reference, the difference in RF conductivity between S-Gr/Cu and P-Cu, as well as M-Gr/Cu and P-Cu, with respect to the frequency variation is analyzed, as shown in Figure 3c. The results in Figure 3c demonstrate that the RF conductivity of M-Gr/Cu obviously decreases compared to P-Cu, whereas the RF conductivity of S-Gr/Cu increases relative to P-Cu. The specific difference in RF conductivity between Gr/Cu and P-Cu is shown in Table 3, represented by $\left(\sigma_{\mathrm{Gr}/\mathrm{Cu}}-\sigma_{P-\mathrm{Cu}}\right)/\sigma_{P-\mathrm{Cu}}$. The results in Table 3 indicate that the difference between P-Cu and M-Gr exceeds 30%, whereas compared to P-Cu, the RF conductivity of S-Gr/Cu increases by more than 15%. These experimental results confirm that Gr can significantly enhance the RF conductivity of Cu but then raise two important questions: (1) what is the mechanism by which monolayer/single-layer Gr enhances RF conductivity; and (2) why does M-Gr/Cu yield the lowest RF conductivity?

### 3.3. The Mechanism of the Difference in RF Conductivity between Gr/Cu and P-Cu

#### 3.3.1. Carrier Concentration and Mobility of S-Gr/Cu and P-Cu in RF Condition

**Figure 4:**
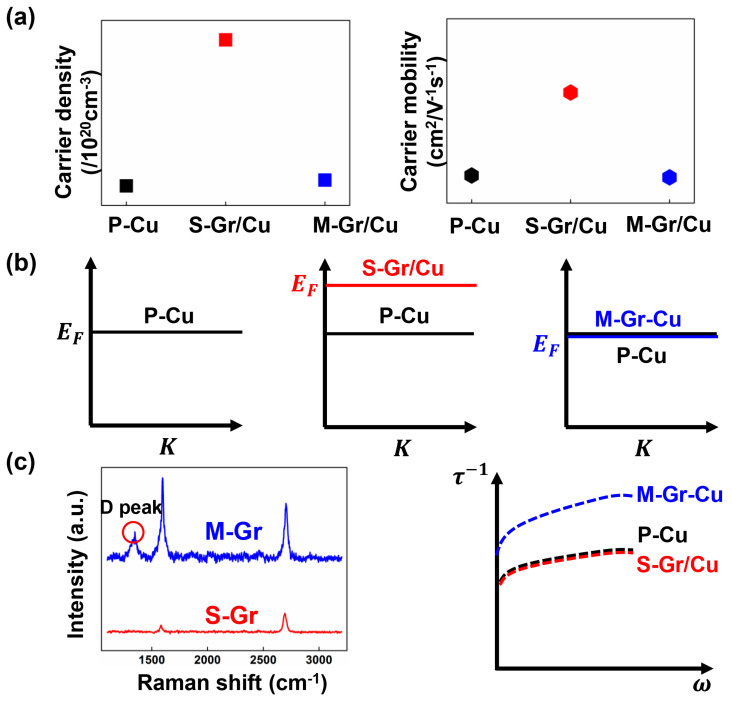
Analysis of the mechanism of conductivity difference among P-Cu, S-Gr/Cu, and M-Gr/Cu at high frequencies: (**a**) comparison of carrier concentration and mobility, (**b**) comparison of Fermi energy levels, (**c**) comparison of Raman results of S-Gr and M-Gr, and comparison of scattering rates of P-Cu, S-Gr/Cu, and M-Gr/Cu.

Firstly, the reasons for the difference in conductivity between S-Gr/Cu and P-Cu under RF conditions are discussed. It is reported that each Gr carbon atom gains approximately 0.24 electrons from neighboring Cu atoms under DC conditions due to the electron doping effect of Cu on Gr. Consequently, the Fermi energy shifts upward by approximately 0.54 eV from the Dirac point, resulting in an electron concentration inside Gr of about 4.3 × 10^14^ cm^−2^, significantly surpassing the intrinsic value of approximately 2 × 10^11^ cm^−2^ for pristine bilayer Gr [13]. Since the conductivity of Gr is positively correlated with the carrier density, the conductivity of Gr increases significantly after electron doping, resulting in a significantly higher conductivity of S-Gr/Cu than that of P-Cu, as shown in Figure 4a.

Different from the DC conditions, the carrier concentration of Gr may be affected by microwave radiation under high-frequency conditions. Tamara Monti utilized near-field microwave microscopy to acquire high-resolution images of CVD Gr and determined that electromagnetic waves induce heating of Gr flakes beneath the apex of the needle’s tip. The corresponding equation for the temperature rise induced by microwave radiation is provided in reference [33]. The rising temperature of Gr by microwave radiation can lead to an elevation in carrier concentration. Moreover, Turja Nandy’s research has demonstrated that variations in the carrier mobility per unit volume in Gr caused by temperature fluctuations result in alterations in the intrinsic carrier concentration across a specific width of Gr, which can be expressed by the following equation:

(6) $$n_{i}=\left(\frac{4}{W_{ac}}\right)\left(\sqrt{\frac{\pi k_{B}T}{E_{G}}}\right)e^{-\frac{E_{g}}{k_{B}T}}$$

The results calculated by Turja Nandy further illustrate that the carrier concentration of Gr exhibits a positive temperature dependence [34]. Based on these results, it can be hypothesized that the transient thermogenesis (~404 K) induced when the high-frequency signal emitted by the tip of the needle interacts with the Gr during the SMIM test process [33,35] results in an elevation of Gr Fermi energy level, leading to the increasing of carrier concentration, as shown in Figure 4b. Considering the significantly higher carrier mobility of Gr and the notably increased carrier concentration of Gr at high frequencies, it can be concluded that $\sigma_{S-Gr/Cu_{ac}}>\sigma_{S-Gr/Cu_{dc}}>\sigma_{P-Cu_{dc}}$ based on the classic Drude model.

#### 3.3.2. Scattering Mechanism of M-Gr/Cu in RF Condition

The degradation of RF conductivity in M-Gr/Cu can be attributed to the presence of defects in the multilayer Gr. Notably, unlike S-Gr, the M-Gr examined in this study exhibits evident defect peaks, as illustrated in the Raman results depicted in Figure 4c. This phenomenon results from the incomplete decomposition of methane under increasing growth pressure, leading to the generation of amorphous carbon atoms within the Gr lattice. These atoms induce Gr lattice vibrations away from the center of the Brillouin zone, resulting in the variations in the electronic character of M-Gr [36]. Theoretical studies have indicated that these defects profoundly impact the energy band structure and density of states of Gr, which in turn affects the scattering state and carrier concentration of Gr carriers. S. Massabeau et al. investigated the temperature dependence of the conductivity of epitaxial multilayered Gr through terahertz time-domain spectroscopy. Their findings suggest that in Gr layers with vacancy defects, the Fermi energy levels are anchored by the mid-gap state near the Dirac point. These vacancy defects induce scattering at short-range potential and mid-gap states [37]. Zakaria Moktadir et al. presents findings on the electron transport behavior of helium-ion-irradiated Gr nanowires. The study reveals that with a defect concentration exceeding 0.3%, the energy band of Gr exhibits a plateau extending throughout the entire region above the Dirac point (n-branch), where the conductivity aligns with the minimum conductivity at the Dirac point. This is primarily attributed to defects generated by helium ion irradiation functioning as charge traps, thereby pinning the Fermi energy levels to the Dirac point [38]. Consequently, this leads to the near disappearance of the n-type doping feature of Gr and no significant enhancement of the carrier concentration regarding electron doping.

These experimental results further confirm that the Fermi energy levels of defect-containing Gr are pinned near the Dirac point. In the context of the present study, the transfer of Cu electrons to Gr, despite potentially occurring due to the pinning of the Fermi energy levels by defects, will not lead to a substantial increase in the Fermi energy level of Gr relative to the Dirac point. Consequently, the differences in conductivity between M-Gr and Cu cannot be discerned by comparing only their carrier concentrations and Fermi energy levels, as depicted in Figure 4b.

Therefore, when comparing the RF conductivity of Gr/Cu with that of Cu, the correlation theory, which posits that conductivity as proportional to the carrier concentration and mobility, is suitable for S-Gr and P-Cu without defects but not for M-Gr and P-Cu with defects. Taking into account the extra scattering resulting from defects, this study evaluates the impact of scattering on conductivity within the framework of the Drude model to analyze the scenario involving M-Gr and P-Cu under RF conditions.

Hanju Lee’s research suggests that in an RF field, the presence of defects within a material leads to significantly higher electric and magnetic field strengths near the defect compared to regions without defects, indicating an inhomogeneity of electric and magnetic fields. This inhomogeneity results in the accumulation of charges or dipoles, referred to as defect dipoles [39]. The impact of defective dipoles on the properties of various materials has been extensively investigated. Zheng et al. suggests that oxygen vacancies in lead zirconate titanate can readily diffuse and combine with dopant ions to form defective dipoles. The formation of these dipoles is a relaxation process gradually induced by the material under a specific external field over time, and they serve as a pinning effect on the electric domains [40]. Huang et al. discovered that the carbon nanotube surface harbors numerous bending and twisting crystal defects, leading to the formation of many electric dipoles. These dipoles serve as dielectric polarization centers under the influence of alternating electromagnetic fields, coupled with the intrinsic differences in dielectric properties of heterogeneous materials, ultimately resulting in the blockade of electron migration and the significant accumulation of electrons at the Cu/C interface, forming heterogeneous interface polarization [41]. Therefore, defective dipoles at the Cu/Gr interface not only impede the movement of carriers but also entrap a certain number of carriers, thereby increasing the likelihood of carrier collisions and resulting in additional carrier scattering. Since DC conductivity measurements are conducted under static conditions, which involve the relaxation of accumulated charge, the effect of defective dipoles can be largely neglected. In comparison, defective dipoles in the RF field lead to additional carrier scattering, as illustrated in the rightmost axis of Figure 4c. Similar situations regarding the influence of dipoles on RF conductivity can be found in previous work of the research group [42]. This could be the primary reason for the significantly lower RF conductivity of defect-containing M-Gr compared to S-Gr and P-Cu.

#### 3.3.3. Gr/Cu Interface-Related RF Conductivity Mechanisms

The aforementioned research indicates that the carrier concentration and mobility of graphene, as well as the scattering caused by defects, significantly affect the RF conductivity of Gr/Cu. In addition to the influencing factors of graphene itself, the interface state of Gr/Cu is also an important factor that cannot be ignored. According to the theory of coherent interface, graphene and polycrystalline copper are non-coherent interfaces, which can cause defects such as dislocations in the copper substrate near the interface. Yang used TEM to observe the interface microstructure between S-Gr and M-Gr and copper and found that due to the existence of dislocations induced by lattice mismatch between Gr and Cu, they inevitably lead to lattice distortion close to the Gr/Cu interface [25]. Therefore, when electrons are transported at the Gr/Cu interface, they will be subject to scattering effects from both graphene and copper substrate defects. However, S-Gr/Cu prepared by chemical vapor deposition is believed to have a Cu (111) orientation, with a lattice mismatch of less than 3% with Gr (0001), which can form a coherent interface. Xiong et al. found through EBSD testing that after chemical vapor deposition of graphene, the Cu substrate changes from polycrystalline copper to a Cu (111) orientation. Combined with PF-AFM mapping method testing, it was found that the conductivity of Gr/Cu and Cu/Cu follows the sequence Cu (111)/Gr/Cu (111) > Cu (100)/Gr/Cu (100) > poly Cu/Gr/poly Cu. This is due to Cu (111)/Gr/Cu (111) having the best lattice match because of the same threefold symmetry and very similar lattice constants between graphene (2.46 Å) and Cu (111) (2.56 Å) [13].

Therefore, for S-Gr/Cu, the formation of a Gr/Cu coherent interface can not only improve the conductivity of the copper substrate but also promote interface bonding between Gr and copper, thereby facilitating electron transfer between Gr and copper and increasing the carrier concentration of graphene. Therefore, the RF conductivity of Gr/Cu is significantly higher than that of pure copper. For M-Gr/Cu, the defects in graphene and the defects in the copper substrate due to lattice mismatch of Gr/Cu can result in severe carrier scattering and significantly reduce the RF conductivity of M-Gr/Cu.

## 4. Conclusions

In summary, the radio frequency (RF) conductivities of copper–copper (P-Cu), monolayer-graphene/copper (S-Gr/Cu), and multilayer-graphene/copper (M-Gr/Cu) structures were examined using scanning microwave impedance microscopy. This study unveiled that the RF conductivity of the prepared Gr/Cu exhibits the following character: M-Gr/Cu < P-Cu < S-Gr/Cu, in contrast to DC conditions where P-Cu < M-Gr/Cu < S-Gr/Cu. Testing of P-Cu and Gr/Cu films was conducted using the dielectric resonator technique, and the same trend was also observed at both 3.1 GHz and 8.2 GHz. The RF conductivity of P-Cu is over 30% higher than that of M-Gr, while the RF conductivity of S-Gr/Cu is over 15% higher than that of P-Cu. In the case of Cu < S-Gr/Cu, the primary disparity between DC and RF conditions lies in the additional thermal effects at the Gr/Cu interface induced by microwave radiation, which further elevate the carrier concentration within Gr compared to DC conditions, resulting in an additional shift of the Fermi level. For the scenario of M-Gr/Cu < P-Cu under RF conditions, the electron doping effect observed under DC conditions fails to account for it. We consider the carrier concentration and carrier mobility of graphene and the scattering effect caused by defects in Gr/Cu as the main factors that affect RF conductivity. The scattering effect of defects includes the scattering of electrons by the defect itself and the polarization loss of the defect dipole caused by non-uniform electric and magnetic fields at the location of defects. The defects in Gr/Cu mainly come from intrinsic defects such as vacancies, grain boundaries, and sp^3^ defects in graphene, as well as defects in the copper substrate near the non-coherent Gr/Cu interface, such as dislocations. Therefore, to further improve the RF conductivity of Gr/Cu, it is necessary to increase the carrier concentration and mobility of graphene and control the type and density of defects in Gr/Cu by preparing high-quality single-crystal Gr and by regulating the lattice mismatch near the Gr/Cu interface. The present results may provide a practical foundation for optimizing the RF conductivity of conductive materials. Further research on the RF conductivity of Gr/Cu in higher frequencies and the evaluation of electromagnetic losses may facilitate the design and fabrication of Gr/Cu multilayers with desired structures and RF conductivity.

## Figures and Tables

**Table 1:** The distribution of ratios of I_2D_/I_G_ in prepared S-Gr and M-Gr.

Type of Gr (Controlled by Growth Pressure)	Ratio of I_2D_/I_G_		
	Percentage with a Ratio of 1.5–2.5 (%)	Percentage with a Ratio of 1–1.5 (%)	Percentage with a Ratio of 0–1 (%)
S-Gr (1 Torr)	98.1	1.3	0.6
M-Gr (250 Torr)	2.1	3.7	94.2

**Table 2:** Electrical conductivity of P-Cu, M-Gr/Cu, and S-Gr/Cu under DC and SMIM conditions.

Test Conditions	Electrical Conductivity (MS/m)		
	P-Cu	M-Gr/Cu	S-Gr/Cu
DC	17 ± 0.8	19.8 ± 1.2	21 ± 1.5
SMIM	17 ± 2.5	6.5 ± 2.2	71.1 ± 2.5

**Table 3:** Comparison of RF conductivity between P-Cu, M-Gr/Cu, and S-Gr/Cu by dielectric resonator cavity testing.

Test Frequency (GHz)	RF Conductivity (MS/m)		
	P-Cu	M-Gr/Cu	S-Gr/Cu
3.1	56.8 ± 1.8	37.1 ± 1.4	65.9 ± 2.9
8.2	51.8 ± 2.5	31.6 ± 2.3	59.8 ± 3.1
$\left(\sigma_{\mathrm{Gr}/\mathrm{Cu}}-\sigma_{P-\mathrm{Cu}}\right)/\sigma_{P-\mathrm{Cu}}$ **(%)**			
	**P-Cu**	**M-Gr/Cu**	**S-Gr/Cu**
3.1	-	−34.68	15.49
8.2	-	−37.06	15.44

## Data Availability

Data are contained within this article.
